# Temporal Profiling of Cellular and Molecular Processes in Osteodifferentiation of Dental Pulp Stem Cells

**DOI:** 10.3390/biology14030257

**Published:** 2025-03-04

**Authors:** Bibiána Baďurová, Kristina Nystøl, Terézia Okajček Michalič, Veronika Kucháriková, Dagmar Statelová, Slavomíra Nováková, Ján Strnádel, Erika Halašová, Henrieta Škovierová

**Affiliations:** 1 Biomedical Centre Martin, Jessenius Faculty of Medicine in Martin, Comenius University in Bratislava (JFM CU), Malá Hora 4C, 036 01 Martin, Slovakia; badurova25@uniba.sk (B.B.); kristina.nystol@so-hf.no (K.N.); tereza.okj@gmail.com (T.O.M.); kucharikova14@uniba.sk (V.K.); slavomira.novakova@uniba.sk (S.N.); jan.strnadel@uniba.sk (J.S.); erika.halasova@uniba.sk (E.H.); 2 Department of Medical Biochemistry, Jessenius Faculty of Medicine in Martin, Comenius University in Bratislava (JFM CU), Malá Hora 4D, 036 01 Martin, Slovakia; 3 Department of Stomatology and Maxillofacial Surgery, University Hospital in Martin and JFM CU, Kollárova 2, 036 01 Martin, Slovakia; dagmar.statelova@uniba.sk

**Keywords:** dental pulp stem cells, osteodifferentiation, calcium deposit production, cell-based therapy, bone tissue engineering

## Abstract

Based on the potential of DPSCs as the most promising candidates for bone tissue engineering, we comprehensively investigated the time-dependent cellular and molecular changes that occur during their osteodifferentiation. To analyze this area in-depth, we used both cellular and molecular approaches. Morphological changes were monitored using bright-field microscopy, while the production of mineral deposits was quantified spectrophotometrically. The expression of a key mesenchymal stem cell marker, CD90, was assessed via flow cytometry. Finally, protein-level changes in whole cells were examined by fluorescence microscopy. Our results show successful long-term osteodifferentiation of the patient’s DPSCs within 25 days. In differentiated cells, mineralized extracellular matrix production gradually increased; in contrast, the expression of the specific stem cell marker CD90 significantly decreased. We observed dynamic changes in intracellular and extracellular proteins when collagen1 A1 and osteopontin appeared as earlier markers of osteogenesis, while apolipoprotein A2, bone morphogenetic protein 9, dentin sialophosphoprotein, and matrix metalloproteinase 8 were produced mainly in the late stages of this process. A decrease in actin microfilament expression indicated a reduction in cell proliferation, which could be used as another marker of osteogenic initiation. Our results suggest a coordinated process in vitro in which cells synthesize the necessary proteins and matrix components to regulate the growth of hydroxyapatite crystals and form the bone matrix.

## Simple Summary

Dental pulp stem cells (DPSCs) stand out as valuable resources for bone tissue engineering due to their easy availability, strong regenerative ability, and ethical advantages over other adult stem cells. However, the exact mechanisms behind their bone-forming potential are not fully understood. This study explores how DPSCs change over time during differentiation, providing new insights into bone matrix formation. With the growing need for better bone regeneration therapies, our findings could benefit regenerative medicine and dentistry. DPSCs offer a biocompatible, patient-specific alternative to traditional bone grafts, reducing surgery risks and improving treatment success.

## 1. Introduction

Mesenchymal stem cells (MSCs) are heterogeneous stem cells and can be isolated from any human tissue; therefore, selecting a suitable cell source is crucial, considering the difficulty and invasiveness of obtaining samples and the acceptance/rejection of cells by allogeneic transplantation [1]. In general, MSCs are characterized by a multidirectional differentiation potential because they are able to transdifferentiate into mesodermal and non-mesodermal cell lines and have a high self-renewal capacity [2,3]. In addition, they are involved in several biological processes, such as immune regulation, neuroprotection, anti-inflammatory, antifibrotic, antioxidant, and angiogenic processes [1,4]. Common criteria for MSCs involve the ability to adhere to plastic under standard culture conditions, the expression of positive (CD73, CD90, and CD105) or negative (CD34, CD45, HLA-DR, CD14, CD11b, CD79a, and CD19) surface markers, and their differentiation capacity for osteoblasts, adipocytes, and chondroblasts in vitro [5].

Teeth are composed of a complex organization of distinct hard and soft tissue types, encompassing highly mineralized structures, such as enamel, dentin, and cementum, as well as soft tissues, including the dental pulp and periodontal ligament [6]. Dental stem cells (DSCs), which can be obtained from dental tissue, are a group of MSCs. Five populations of DSCs were chronologically discovered: dental pulp stem cells (DPSCs) [7], stem cells from exfoliated deciduous teeth (SHEDs) [8], periodontal ligament stem cells (PDLCs) [9], stem cells from the apical papilla (SCAPs) [10], and dental follicle precursor cells (DFPCs) [11]. Based on many results, DPSCs appear to be a promising cell source for stem cell-based therapy [12,13,14]. DPSCs not only offer the advantage of low immunogenicity compared to other sources of MSCs or progenitor cells but are also ethically uncontroversial and easy to access. Moreover, DPSCs can be harvested without invasive procedures or risk to the donor [1,15]. They are easily isolated from the third molars of deciduous or adult teeth [16], which are usually extracted for health reasons and then disposed of as biological waste. Since a person has two sets of teeth during his life, a large amount of DPSCs, which are currently waste materials, can be used as an excellent source of stem cells in regenerative medicine in the future [17]. DPSCs originate from the neural crest of the brain and are capable of neural-, adipo-, and chondrodifferentiation [18,19,20]. However, their osteogenic potential is the most promising. Compared to commonly used bone marrow mesenchymal stem cells (BMMSCs), DPSCs have faster population doubling and significantly higher alkaline phosphatase (ALP) activity, supporting their use for mineralized tissue regeneration [21].

The ability of stem cells to differentiate into osteogenic lineages is an important part of bone tissue regeneration. The human skeleton has many functions, such as mechanical support of the body, movement ability, protection of organs, blood cell formation, and storage of minerals [22]. Despite the ability of bone to regenerate and repair itself, approximately 5–10% of patients suffering from bone fractures may experience healing failure. Successful bone healing requires the co-presence of three essential processes: osteoinduction, osteogenesis, and osteoconduction. Osteogenesis is the developmental process through which stem cells commit, proliferate, and differentiate to an osteoblastic lineage and begin to produce extracellular matrix (ECM), resulting in bone formation [23]. The bone matrix comprises inorganic (60%) and organic compounds (40%). The organic phase, which consists mainly of collagen type I (90%) and non-collagenous mineral-binding proteins (NCPs) (10%), has a much lower density, stiffness, and strength than the inorganic phase, which is dominated by hydroxyapatite [24]. During the complex process of osteogenic differentiation, a variety of markers associated with osteogenic differentiation are expressed, such as alkaline phosphatase (ALP), bone morphogenetic proteins (BMPs), collagen type 1 (COL1), osteocalcin (OCN), osteopontin (OPN), dentin matrix protein 1 (DMP1), dentin sialophosphoprotein (DSPP), stromal extracellular phosphoglycoprotein (MEPE), and others [25,26].

DPSCs have great potential in stem cell-based therapies, mostly in hard tissue regeneration [27]. Therefore, the main objective of the present work was to study the osteodifferentiation process of DPSCs isolated from the patient, using various approaches in a time-dependent manner, including on a (i) biological level to monitor and visualize changes in cell morphology, (ii) biochemical level to quantify mineral deposit production, and (iii) molecular level to analyze protein production in control and differentiated cells to obtain more comprehensive knowledge about their abundance. Although there are publications in which the authors have monitored DPSCs after osteogenic differentiation [28,29], we have not yet encountered complex studies on the long-term osteodifferentiation of DPSCs in which analyses were performed every 5 days for a 25-day period. Furthermore, results on monitoring intra- and extracellular protein expression to the extent presented in the current paper are not available in the literature.

## 2. Materials and Methods

### 2.1. Isolation, Cultivation, and Differentiation of Dental Pulp Stem Cells

Adult human MSCs derived from dental pulp (DPSCs) were isolated according to our optimized protocol [30]. Original cell lines were generated from two various donors and used in this study. Cells were grown in a DMEM/F12 + GlutaMAX (Gibco, Grant Island, NY, USA) culture medium supplemented with 10% FBS (Gibco), 100 U/mL penicillin, and 100 µg/mL streptomycin (Biosera, Cholet, France) and cultured under standard cultivation conditions (5% CO_2_, 37 °C, and humidified conditions). DPSCs were seeded at a density of 5 × 10^3^ cells/cm^2^. When cells reached 60% confluence, they were differentiated with commercially available osteodifferentiation medium OsteoMAX-XF (Merck, Darmstadt, Germany) supplemented with 100 U/mL penicillin and 100 µg/mL streptomycin (Biosera). Unexposed controls were prepared simultaneously. The culture medium was exchanged every 3rd day so that 100% of the medium was exchanged in control cells and 25% in differentiated cells. Cells were processed for individual analyses on the 5th, 10th, 15th, 20th, and 25th days from the beginning of osteodifferentiation. The following analyses were performed on passages 3–5.

### 2.2. Monitoring of Cell Morphology

The cell morphology of the control and osteodifferentiated DPSCs was monitored throughout the experiment. Bright-field photomicrographs were captured by light microscope (Optika IM-3, Ponteranica, Italy) on the 5th, 10th, 15th, 20th, and 25th days after the beginning of osteodifferentiation.

### 2.3. Alkaline Phosphatase Staining

ALP staining was performed using an AP staining kit (System Biosciences, Palo Alto, CA, USA) following the method. Briefly, cells were washed and fixed with a fixative solution (from the kit) for 3 min at room temperature (RT). After washing 1× with DPBS (Dulbecco’s phosphate-buffered saline, Biosera), cells were incubated for 20 min at RT in freshly prepared AP substrate solution (1:1 prepared from solution A:B). Subsequently, cells were rinsed 2× with DPBS, kept in DPBS, and photomicrographed using a light microscope (Optika IM-3).

### 2.4. Alizarin Red Staining and Quantification of Calcium Deposits

In this assay, control and osteodifferentiated cells (*n* = 6/group) were washed 3 times with DPBS, followed by fixation in 4% PFA (Cell Signaling Technology, Danvers, MA, USA) for 30 min at RT, dark. After washing 3× with DPBS, 2% Alizarin Red S (Merck) was added to each well for 3 min. The dye was removed, and the stained deposits were carefully washed with ddH_2_O and visualized using a light microscope (Optika IM-3). Then, for quantifying the calcium deposits, destain (10% Acetic Acid (Serva, Heidelberg, Germany) + 20% Methanol (Serva) + 70% ddH_2_O) was added to each well for 15 min at RT with slight shaking. The solution was transferred to tubes and centrifuged at 10,000 rpm at 15 °C for 10 min to remove the insoluble deposits. Subsequently, the absorbance of individual samples was read at OD 405 nm using an Epoch microplate reader (BioTek, Winooski, VT, USA). The protein concentration was determined by the BCA assay (Pierce BCA Protein Assay Kit, Rockford, IL, USA). Cells were washed 1× with DPBS and then incubated with RIPA buffer (Sigma-Aldrich, St. Louis, MO, USA) supplemented with protease inhibitor (Roche, Mannheim, Germany) for 5 min at RT. The cells of the wells were scraped, transferred to microtubes, sonicated for 2 × 10 sec on ice, and followed by centrifugation at 12,000 rpm, 8 °C for 20 min. The protein concentration was measured in supernatants along with BSA prediluted standards (Pierce). After 30 min of incubation at 37 °C, the absorbance was measured at OD 562 nm using the Epoch microplate reader (BioTek).

### 2.5. Flow Cytometry

The expression of CD90, a typical marker of MSCs, was analyzed by flow cytometry. Control and osteodifferentiated cells (*n* = 6/group) were passaged, and a cell pellet was resuspended in DBPS. Cells were strained in a 70 µm cell strainer (Corning, Durham, NC, USA) to prepare a single-cell suspension. This suspension was centrifuged at 450× *g*, 13 °C for 4 min, and the cells were resuspended in FACS buffer (5% mouse serum (Sigma-Aldrich) in DPBS and 1 mmol/l of EDTA (Gibco)) and counted. After blocking for 30 min in the dark and on ice, marker CD90-APC (BioLegend, San Diego, CA, USA) was incubated with cell samples for 60 min in the dark and on ice. Consequently, the labeled samples were washed by DPBS, followed by centrifugation at 450× *g*, 13 °C for 4 min, and the stained cell pellet was resuspended in FACS buffer. The samples were analyzed by BD FACSDiva software v8.0.1 on a flow cytometer (BD Biosciences FACS Aria, San Jose, CA, USA). Analysis was performed on 10,000 events, and the gating was set up based on an unstained/isotype control, while the gating in all samples at each time interval was consistent.

### 2.6. Immunocytochemistry

Cells grown in dark 96-well plates (Sarstedt, Numbrecht, Germany) were washed with DPBS and fixed in 4% PFA (Cell Signaling Technology) on the 5th, 10th, 15th, 20th, and 25th days from the beginning of osteodifferentiation. The cells were fixed for 30 min at RT in the dark. After washing, the cells were incubated with specific antibodies. Samples stained with Alexa Fluor^®^ 488 Phalloidin (Abcam, Cambridge, UK, ab176753, 1:1000 in a solution of 1% BSA in DPBS) were incubated for 80 min. Consequently, the samples were labeled with DAPI dye (Sigma-Aldrich) for 10 min at RT in the dark. The cells were washed 3 times with DPBS and analyzed using a fluorescence microscope (WiScan^®^, Hermes IDEA Bio-Medical, Rehovot, Israel). Samples stained with specific primary antibodies APOA2 (Abcam, ab92478, rabbit, 1:200), BMP9 (Invitrogen, Waltham, MA, USA, PA5-28816, rabbit, 1:100), COL1A1 (Cell Signaling Technology, 39952, rabbit, 1:500), DSPP (Invitrogen, PA5-76382, rabbit, 1:200), MMP8 (Sigma-Aldrich, HPA022935, rabbit, 1:100), and OPN (Sigma-Aldrich, HPA027541, rabbit, 1:100), washed 3 times with DPBS, and incubated in blocking solution (5% goat serum (Sigma-Aldrich) + 0.2% Triton X-100 (Sigma-Aldrich) in DPBS) for 60 min in the dark at RT. The samples were incubated with primary antibodies in a blocking solution overnight at 4 °C. After washing 3× with DPBS, they were incubated with a fluorescent secondary antibody (Abcam, ab150077, antirabbit, 1:500) in a blocking solution for 60 min in the dark at RT. Consequently, the samples were labeled with 1 µg/mL DAPI dye (Sigma-Aldrich) for 10 min at RT in the dark. Cells were washed 3 times with DPBS and analyzed using a fluorescence microscope (WiScan^®^). Negative controls were included for all samples, where cells were stained exclusively with the fluorescent secondary antibody, omitting the primary antibodies. These controls confirmed the absence of the autofluorescence of the cells. Semiquantitative analysis of protein expression was conducted using Fiji/ImageJ software v1.8.0_345 as follows: The channels in all samples were separated, resulting in two distinct color channels displayed in separate windows, enabling independent quantification of each fluorescent channel. Initially, cell nuclei were counted (blue channel), with a consistent threshold applied across all samples. Subsequently, the expression level of the specific protein was quantified (green channel), maintaining a uniform threshold for all samples stained with the same antibodies at each time point [31]. The expression intensity of each sample was normalized to its nucleus count, and the results were analyzed in Excel (version 1808), with the values quantified as a relative ratio of differentiated cells to control cells (*n* = 6/group).

### 2.7. Statistical Analysis

For statistical analysis of mineral deposit quantification and changes in surface marker expression and protein abundancy, a two-tailed Student’s *t*-test (pooled variance) was evaluated using Statistic Kingdoms software (https://www.statskingdom.com/140MeanT2eq.html; accessed on 14 November 2024) at adjusted *p* ≤ 0.5 (*), *p* ≤ 0.05 (**), and *p* ≤ 0.005 (***). Excel (version 1808) was used to prepare data for statistical analysis to quantify differentially abundant intra/extracellular proteins. Data are presented as the mean ± SD (*n* = 6/group). All experiments were evaluated in three replicates.

## 3. Results

### 3.1. Differentiation of DPSCs into Bone Cells

**Figure 1:**
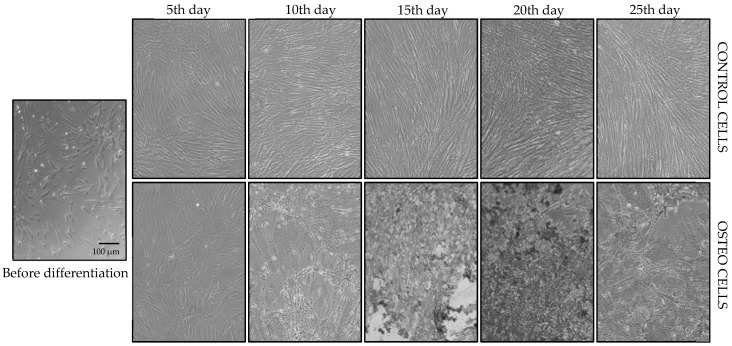
Cell morphology changes and cell density monitored by light microscopy before and during osteodifferentiation of DPSCs over 25 days. Control cells were cultivated in standard basal medium and differentiated cells in osteodifferentiation medium, which induced calcium deposit production in cells.

In this study, we monitored the osteodifferentiation process of patients’ DPSCs in a time-dependent manner over 25 days. We studied the ability of these stem cells to differentiate into osteoblasts and mature osteocytes. Simultaneously, these cells were grown in a DMEM/F12 + GlutaMAX cultivation medium (control cells) and an osteodifferentiation culture medium OsteoMAX-XF (osteodifferentiated cells), which we chose based on our previous studies [30,32]. Cell morphology was captured on the 5th, 10th, 15th, 20th, and 25th days of this process (Figure 1).

DPSCs, as MSCs, showed spindle-shaped and fibroblast-like morphology, reached full confluence, and had high proliferation activity in the primary cell culture. Early morphological changes were detected on the third day of differentiation. Differentiated cells started to be shorter and octagonal-shaped compared to control cells, which retained fibroblastic-like morphology. Furthermore, osteodifferentiated cells stopped their proliferation activity, with a total of 3.13 × 10^5^ differentiated cells compared to 8.11 × 10^5^ control cells on the 5th day of differentiation. For the rest of the time, we were not able to count the differentiated cells due to mineral deposit interaction during the counting process. On the 10th day, differentiated cells started to produce an ECM containing calcium deposits, which almost covered the cells completely. Over time, there was a gradual reduction in the cell population. During osteodifferentiation, cells formed a heterogeneous population. On the 25th day, some cells differentiated into osteocytes, while others remained osteoblasts. Additionally, we observed long fibrous cells, which we hypothesize to be either original MSCs or residual cells from the biological sample, contributing to the newly generated DPSC line.

### 3.2. Time-Dependent Production of Calcium Deposits in Differentiated Cells

**Figure 2:**
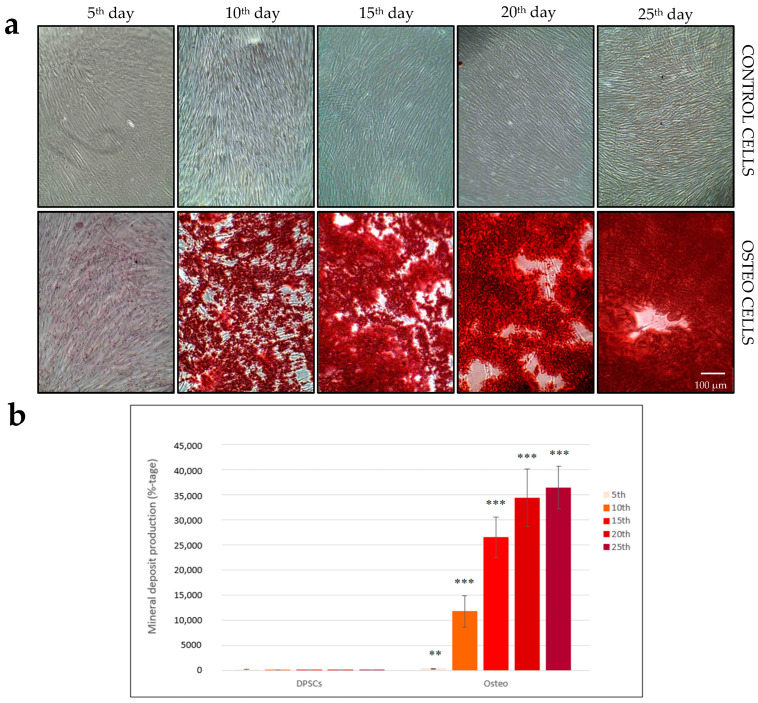
Alizarin Red staining of calcium deposits of both control (DPSCs) and osteodifferentiated (Osteo) cells in a time-dependent manner over 25 days from the beginning of osteodifferentiation. (**a**) The stained mineral deposits were visualized by light microscopy; (**b**) Mineral deposit quantification was measured by a spectrophotometer. The graph shows an increase in calcium deposit production over 25 days. Gradual increase in calcium compounds in osteodifferentiated cells confirms bone matrix production. Control cells have a value of 100% at all time points. The values are the mean ± SD control and osteodifferentiated cells (*n* = 6). The statistical significance of the change between control and differentiated cells on each day is represented by the *p*-values *p* ≤ 0.05 (**) and *p* ≤ 0.005 (***).

Subsequently, we performed immunohistochemical staining with Alizarin Red, which binds to calcium compounds, to confirm their presence. It indicates successful osteodifferentiation of stem cells into osteoblasts and the dynamics of their production in a time-dependent manner. The control cells remained unstained after washing with Alizarin Red dye. Osteodifferentiated cells showed deep red staining, which revealed the presence of a large number of calcium deposits in the ECM (Figure 2a).

We quantified their amount in osteodifferentiated cells in relative relationship to control DPSCs. On the 5th day, the increase reached 2.8 times, but the most substantial changes occurred on the 10th day with a 117-fold increase and on the 15th day with a 264-fold increase. On the 20th and 25th days, there was only a slight increase in the number of calcium compounds, with a 343-fold increase on the 20th day, while on the final 25th day of this process, there was a 363-fold increase in differentiated cells compared to controls (Figure 2b).

### 3.3. Temporal Variation in ALP Production

The ALP enzyme is considered an early marker of osteoblast differentiation because it is produced as a by-product of osteoblast activity [33]. We used ALP staining as further confirmation of stem cell differentiation into osteoblasts/osteocytes. The increase in the expression of ALP is visible as a blue coloration, which is a product of the chemical reaction that ALP catalyzes. The most significant expression of ALP in osteodifferentiated cells was observed on the 5th day of the process. On the 10th day, the cells produced a substantial amount of ECM, making it difficult to observe the cells clearly. However, the expression remained detectable in cells. Control cells did not express ALP on the 5th day However, from the 10th day, we observed a partial blue coloration, but it did not copy the exact morphology of the cells. Therefore, we assume that there may be a nonspecific binding of the dye in the controls. The results from ALP staining are included in Figure S1.

### 3.4. Reduction in MSCs Marker Expression in Osteodifferentiated Cells

Based on previous immunohistochemical staining, we decided to follow the progression of osteodifferentiation by analyzing the expression of CD90, a surface-specific marker of the stem cell phenotype, using flow cytometry. It was performed on control and osteodifferentiated cells on the 5th, 10th, 15th, 20th, and 25th days of this process using a fluorescently labeled antibody against the CD90 marker.

**Figure 3:**
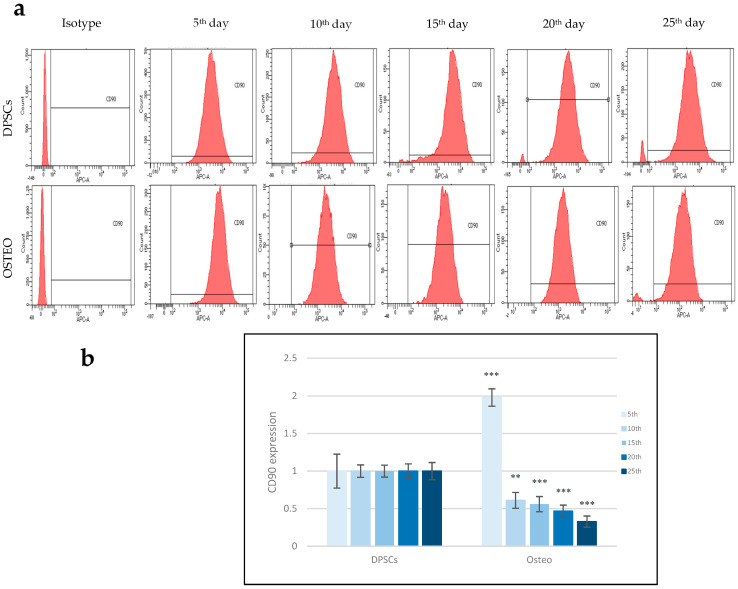
Flow cytometry analysis of control (DPSCs) and osteodifferentiated cells (Osteo) over 25 days. (**a**) Histograms show expression of CD90, a surface-specific marker of stem cell phenotype, in both types of cells; (**b**) The graph shows a decrease in CD90 expression in osteodifferentiated cells over a period of 25 days. Control cells have a value of 1 at all time points. The values are the mean ± SD control and osteodifferentiated cells (*n* = 6). The statistical significance of the change between control and differentiated cells on each day is represented by the *p*-values *p* ≤ 0.05 (**) and *p* ≤ 0.005 (***).

The level of expression was stable in control DPSC cells at all time points for 25 days. However, the CD90 fluorescence intensity in osteodifferentiated cells decreased with time, showing the leftward shift of the peak on the histogram (Figure 3a). CD90 expression was quantified as the relative ratio of differentiated cells to control cells, where the fluorescence intensity of differentiated cells was normalized to the controls on the corresponding day. The control was assigned a value of 1 across all time points. The graph illustrates a significant continuous decrease in CD90 expression, which begins on the 10th day of differentiation (Figure 3b).

### 3.5. Immunochemical Monitoring of Differentially Abundant Osteoproteins in Time-Dependent Manners

**Figure 4:**
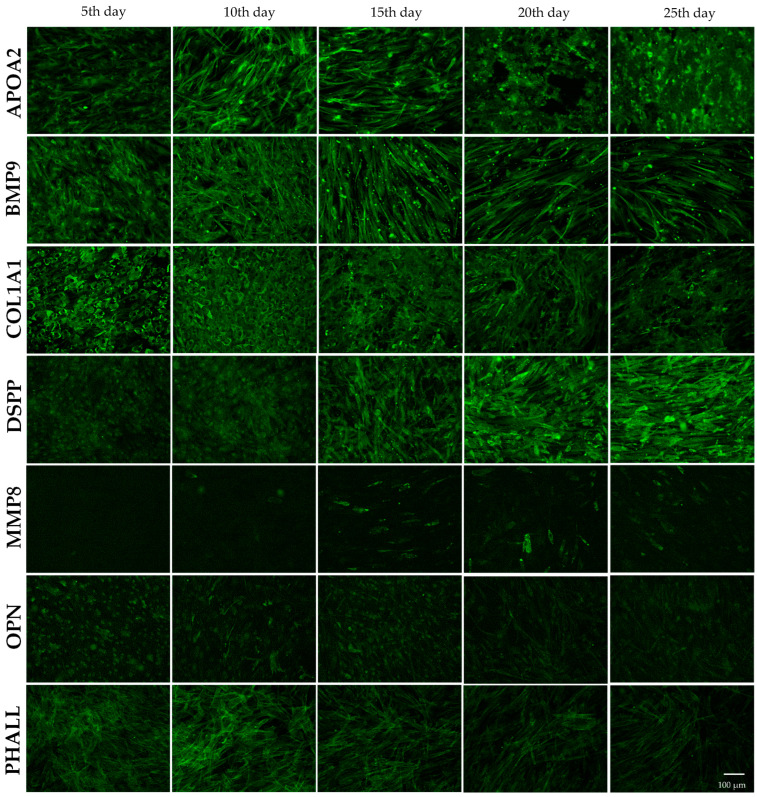
Immunocytochemical staining of osteogenesis markers was performed using fluorescent antibodies over 25 days following the initiation of osteodifferentiation in dental pulp stem cells (DPSCs). Both control and osteodifferentiated cells were stained on the 5th, 10th, 15th, 20th, and 25th days with specific fluorescent antibodies against apolipoprotein A2 (APOA2), bone morphogenetic protein 9 (BMP9), collagen1 A1 (COL1A1), dentin sialophosphoprotein (DSPP), matrix metalloproteinase 8 (MMP8), and osteopontin (OPN). Actin filaments of the cytoskeleton were labeled with an antibody against phalloidin (PHALL). This figure represents the osteodifferentiated cells, while images of the control cells are shown in Figure S2. Fluorescent signals were captured and analyzed using fluorescence microscopy.

In previous analyses, we confirmed the ongoing osteodifferentiation of DPSCs into osteoblasts and osteocytes [30,32]. Subsequently, we wanted to monitor their expression at different time points and determine whether they are early or late osteogenesis markers of DPSCs in our conditions using immunocytochemical staining. Cells were stained every 5 days during osteodifferentiation with specific fluorescent antibodies against osteogenic proteins apolipoprotein A2 (APOA2), bone morphogenetic protein 9 (BMP9), collagen1 A1 (COL1A1), DSPP, matrix metalloproteinase 8 (MMP8), and OPN (Figure 4 and Figure S2).

Also, the progression of osteogenesis in differentiated cells is indicated by a reduction in phalloidin expression, which binds to actin filaments in the cell cytoskeleton. This decrease corresponds to a notable decline in cell proliferation. The localization of all proteins in the differentiated cells was altered compared to control cells. APOA2 and COL1A1 proteins translocated from the intracellular space into the ECM in differentiated cells, forming a cross-linked structure on the 25th day. Moreover, we observed a higher expression of APOA2; in contrast, the expression of COL1A1 was decreased. The BMP9 protein had a relatively high level of expression throughout the osteodifferentiation process and showed significantly higher abundance on the 20th day compared to control cells. DSPP appears to be a later marker of osteodifferentiation in our conditions, as its expression gradually increased from the 5th day. MMP8 expression increased significantly from the 10th to the 25th day. In contrast, OPN had the most significant decrease in its expression in the first 15 days and was located in the nucleus area before translocating to other parts of the cells over time, but its expression was similar to control cells. The results in Figure 4 represent osteodifferentiated cells.

**Figure 5:**
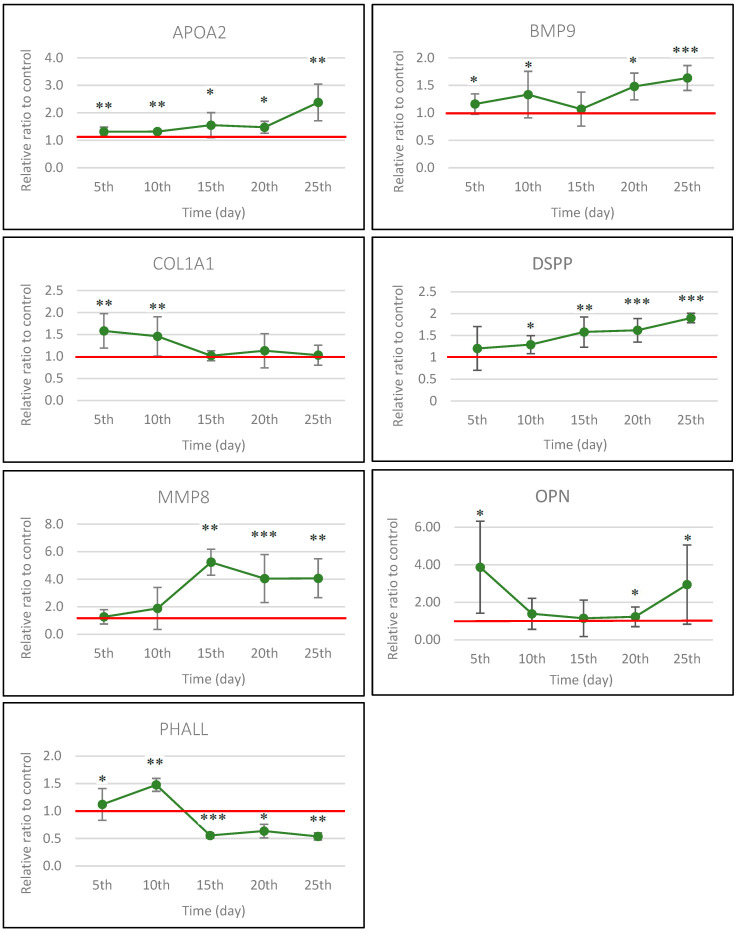
The graphs provide a graphical representation of the abundance of specific proteins, apolipoprotein A2 (APOA2), bone morphogenetic protein 9 (BMP9), collagen1 A1 (COL1A1), dentin sialophosphoprotein (DSPP), matrix metalloproteinase 8 (MMP8), osteopontin (OPN), and phalloidin (PHALL), which binds to actin filaments. The graphs represent the quantification of results from immunocytochemical analysis conducted over 25 days following the initiation of osteodifferentiation in DPSCs. The data are presented as a relative ratio to control cells, with control cells assigned a value of 1 (red line). The values are the mean ± SD osteodifferentiated vs. control cells (*n* = 6). The statistical significance of the change between control and differentiated cells on each day is represented by the *p*-values *p* ≤ 0.5 (*), *p* ≤ 0.05 (**), and *p* ≤ 0.005 (***).

The visualization of control cells with/without DAPI staining is shown in Figure S2. We quantified the expression levels of all proteins (see Section 2.6) and displayed them in graphs (Figure 5), while the values were quantified as a relative ratio of differentiated cells to control cells.

## 4. Discussion

Our study investigated the morphological characteristics and osteogenic/odontogenic differentiation of DPSCs during prolonged incubation. Distinguished osteogenesis from odontogenesis in vitro is a great challenge, if almost impossible. When stem cell maturation begins, no specific or individual markers are available to clearly separate these two processes. However, both pathways might be able to work together in DPSCs under in vitro cultivation. Accordingly, we discussed the process of osteogenesis and focused on delineating the temporal progression of DPSC maturation into bone cells. Our results indicate that osteogenesis is a highly dynamic process, including cell remodeling and migration, production of the ECM accumulating above the cells, and osteocyte embedding. Osteoblasts play a crucial role in the formation of new bones. They progress through three key stages of differentiation: osteoprogenitor cells, preosteoblasts, and mature osteocytes. These cells develop from MSCs through a series of complex differentiation pathways. In the early stages, MSCs within osteogenic lineages differentiate into osteoprogenitor cells, followed by osteoblasts, and can further differentiate into osteocytes, which are terminally differentiated cells [34,35,36]. Therefore, we monitored the maturation of DPSCs into bone cells at various levels, e.g., cytological (monitoring cell morphology and density, as well as the generation and accumulation of mineral deposits in differentiated cells), biochemical (quantification of produced calcium compounds in the ECM), and molecular (studying variation in the protein intra- and extracellular abundance).

Based on our previous results, we decided to use the OsteoMAX-XF differentiation medium (composition is confidential) in our experiments [30,32]. Changes in cell morphology occurred on the 3rd day when cells became shorter and octagonal/polygonal in shape compared to control cells and remained to show fibroblast-like morphology. Differentiated cells slowed their mitotic activity, with a focus on differentiation. In addition, the total amount of differentiated cells decreased more than twice. Control cells did not change their morphology during prolonged incubation. DPSCs have been reported to maintain an undifferentiated state even after long-term cultivation [37] and be little influenced by the number of passages [38]. Analyzing the morphology of the differentiated cells was quite complicated due to increased ECM production, which overcrowded them. We observed that the number of differentiated cells decreased rapidly (Figure 1). It was noticed that osteoblasts undergo apoptosis during maturation into osteocytes, which is part of normal development and tissue homeostasis [29,39]. Park et al. found that p53 signaling is important for the osteogenic differentiation of human periosteal-derived cells [40]. The tumor suppressor p53, a pivotal transcription factor with sequence specificity, governs the expression of various direct target genes involved in cell cycle arrest and activation of apoptotic pathways [41,42]. Inhibition of protein p53 diminished the extent of osteoblast differentiation. Recent studies have demonstrated that the PI3K/AKT/MDM2 axis regulates bone homeostasis by selectively activating numerous signaling molecules through the PI3K/AKT pathway in bone tissue, particularly in osteoblasts [43].

An easily quantifiable marker of osteogenic differentiation is the synthesis of the ECM. We monitored the formation of the calcium deposits by Alizarin Red staining, followed by light microscopy. The matrix was produced above and attached to the cells, becoming denser with prolonged cultivation. Differentiated cells started to produce mineral deposits on the 5th day. Its quantity progressively increased with the duration of the cultivation. We quantified the amount of calcium deposits. The abundant mineralization with high calcium content began on the 10th day, with an almost 117-fold increase compared to control cells. Production continued during prolonged differentiation and increased more than 264 times on the 15th day and up to 363 times on the 25th day in osteodifferentiated cells compared to the control cells (Figure 2). Most of the research provided qualitative results without any time-dependent changes normalized to the total number of proteins [44]. On the other hand, various protocols and compositions of osteodifferentiation media have been used. Mostly, osteodifferentiation media comprises ascorbic acid, β-glycerolphosphate, dexamethasone, and optionally, 1,25-dihydroxycholecalciferol (vitamin D3). One of the limited factors is the serum concentration and its origin. Most of the research had been performed with 1–20% animal fetal/calf serum [2,45,46,47,48]. High concentrations of FBS (20%) alone have been documented to induce the osteogenic differentiation of DPSCs under extended cultivation conditions. Therefore, decreasing the concentration of FBS is preferable to reduce the effect of serum unidentified components on osteodifferentiation [47]. OsteoMAX-XF, used in the study, is prepared without animal sera but with 10% osteogenic supplements. Compared to other commercially available or laboratory-prepared differentiation media, it has rapid induction/activation of osteogenesis in DSCs [30].

We also examined the production of ALP, which is one of the most typical early markers of osteogenesis. ALP production was significantly elevated on the 5th day in differentiated cells compared to control cells. These colorimetric changes were monitored microscopically. On the 10th day, when there was a marked increase in ECM production, a distinct difference in the assessment of the color change for ALP activity was quite problematic to observe (Figure S1). This was due to the overlapping of calcium deposits with the cells and a noticeable concurrent reduction in cell numbers. There are some contradictory findings on the relationship between cell proliferation and ALP activity in the literature [49,50]. Elevated ALP activity may correspond to an increasing number of osteoblasts; however, cell proliferation decreased during differentiation. Consequently, ALP activity decreases as mineralization occurs, which aligns with our observations [51].

Next, we investigated the expression of the surface marker CD90 (Figure 3). This membrane protein is one of the typical markers for MSCs and is highly expressed in DPSCs [52,53]. CD90, also known as Thy1, is a glycosylphosphatidylinositol (GPI) anchored glycoprotein that promotes osteoblast differentiation [54]. CD90 knockdown has been demonstrated to modulate the stemness of MSCs, promoting a shift toward an enhanced propensity for differentiation. Flow cytometry analysis is important in quantitatively evaluating protein expression and differentiation efficiency. We proved the decrease in the expression and distribution of this surface marker during differentiation. On the 5th day, CD90 expression increased. Even differentiated cells underwent cellular morphological changes; we detected an almost 2-fold increase in CD90 distribution in differentiated cells compared to control cells. However, on the 10th day, the expression started to decrease, and from the 15th day (a 2-fold decrease), this continued to the 25th day (more than a 3.3-fold decrease). All of these measurements showed statistically the most significant changes, which point to an active remodeling of plasma membrane protein composition and elimination of stem markers in differentiated cells.

To obtain a more complex view of the dynamic of each studied protein, we analyzed its production in time from the 5th up to the 25th day (Figure 4). To our knowledge, this is the first study to comprehensively examine the molecular and cellular dynamics involved in osteogenesis, particularly focusing on the behavior of various proteins under specific experimental conditions. This investigation offers new insights into the intricate molecular processes that drive osteogenic differentiation, which have not been explored in previous research. Each of the selected proteins is associated with osteodifferentiation. The expression profile of key osteogenic markers shows considerable variation over time (Figure 5).

The dysregulation of lipid metabolism affects bone homeostasis, leading to bone mass and quality changes. An association between bone mass and high-density lipoprotein (HDL) has also been suggested. APOA2, the second most prevalent HDL protein in vertebrates, promotes cholesterol efflux and increases when apolipoprotein A1 (APOA1) is absent [55]. It is involved in metabolic processes, such as maintaining plasma HDL, modulating insulin resistance, and contributing to weight gain [56]. Histological analyses have shown that mature osteoblasts and osteoblast progenitors contain stored lipids. Furthermore, both in vivo and in vitro studies have confirmed that osteoblasts take up circulating lipoproteins and free fatty acids [57]. We identified the accumulation of APOA2 in osteodifferentiated cells using a proteomic approach [32]. In the present study, APOA2 production was evident from the 5th day, conforming to the cell’s shape and displaying a delicate fibrous structure. From the 15th day, the fluorescence intensity increased, and the signals began to spread from the intracellular space to the ECM. On the 25th day, the protein was the most abundant present, predominantly within the ECM, where it overlapped with osteodifferentiated cells in certain areas.

BMPs have substantial therapeutic potential for bone and musculoskeletal disorders, which can be attributed to their osteoinductive capabilities. More than 20 BMPs have been identified in humans, each contributing to embryonic development, skeletal formation, hematopoiesis, and neurogenesis. BMPs facilitate MSC differentiation into osteoblasts and stimulate the proliferation of osteoblasts and chondrocytes [40,58]. Recent research has identified BMP9 as the most potent inducer of osteogenic differentiation in MSCs, both in vitro and in vivo, although its specific role in the skeletal system remains unclear [59,60,61]. BMP9 activates p53 proteins, which initiate apoptosis through the PI3K/AKT/MDM2 cascade in cells, thus supporting bone homeostasis [40]. We obtained abundant BMP9 production from the 5th day. The protein was predominantly localized within the intracellular space, and on the 15th day, we observed the formation of fine fibrous structures. Furthermore, there were intense small signals, which were likely being captured and incorporated into the ECM. The number of cells showed a significant reduction on the 20th and 25th days compared to the 5th day, indicating a marked decrease in cellular density over time, corresponding to the almost linear increase in BMP9 activity during prolonged osteodifferentiation.

ECM protein secretion and mineralization are key traits of mature secretory osteoblasts. Our results show that differentiation media upregulated essential proteins to establish a functional ECM. The abundant production of COL1A1 was significantly elevated on the 5th day and localized within preosteoblasts, which exhibited an octagonal morphology in contrast to the spindle-shaped control cells. On the 10th day, the protein began to be distributed into the extracellular space, contributing to newly synthesized ECM-forming structures. The differentiation media also increased the amount of the mineralized matrix. Despite this extracellular deposition, high levels of intracellular protein persisted. From the 15th day onward, the expression levels decreased. Fluorescence analysis reveals that the protein was primarily within the ECM. This mature protein, a predominant component of a bone ECM, remained a stable marker and the most abundant protein in the newly generated ECM. Additionally, the cells remodeled the collagen fiber network, resulting in the formation of holes corresponding to the early lacunae. Similarly, the physical restructuring of the collagen matrix was evident, with the development of future osteocyte lacunae that appear to be facilitated by cells that displace collagen fibers outside the center of the emerging lacunae [62,63]. Our results provide a new insight into the dynamics of extracellular matrix formation in osteodifferentiated human cells in vitro, similar to Shifflet et al., who highlighted collagen dynamics in osteocytes during mineralization in mice [62].

DSPP is known to be the most abundant non-collagenous protein, which participates in the matrix mineralization process of dentinogenesis and osteogenesis [64]. Furthermore, Figueredo et al. proved that DSPP is an essential protein for the normal mineralization of craniofacial tissues [65]. It may be misleading to consider DSP as an exclusive specific expression of dentin tissue [64]. In our previous work, we detected DSP accumulation in osteodifferentiated cells [32]. We found a continuous increase in its production up to the 25th day. It started on the 5th day and increased almost linearly during prolonged cultivation. In addition, we were able to detect the protein in the cytoplasm, and from the 20th day, a small amount was exported as part of the ECM. Our results about the DSPP translocation correspond to the observation of Baldión et al., who focused their work on odontoblast-like cells. They detected the secreted DSP protein outside cells as part of the ECM after 21 days of treatment [48].

Matrix metalloproteinases (MMPs) function not only as enzymes but also as key degradative agents essential for the regeneration of the ECM, including the regeneration of bone tissue. Furthermore, they are included in various cellular processes, such as cell–cell adhesion, tissue remodeling, cell proliferation, migration and invasion, and apoptosis [66]. MMP8 plays a pivotal role in both normal physiological processes and pathological conditions, including cardiovascular, musculoskeletal, renal, digestive, and respiratory disorders, as well as cancer [67]. MMP8 is involved in ECM remodeling during tissue repair and wound healing. This metalloproteinase acts as an important enzyme in protein degradation and the hydrolysis of peptide bonds [68]. MMP8 digests collagens I, II, III, VII, and X, with a distinct preference for collagen type I, followed by type III and type II. Interestingly, it degrades heterotrimeric collagen type I approximately 20 times more efficiently than homotrimeric collagen type III, emphasizing its critical role in maintaining ECM dynamics [67,69]. We observed a marked increase in the expression of the MMP8 protein from the 15th day onward, aligning with the period when osteodifferentiated cells exhibit robust ECM production. Elevated levels of MMP8 closely correspond to the progressive accumulation of the ECM, suggesting a potential role for MMP8 in the regulation or remodeling of the matrix during this stage of osteogenesis.

OPN is an ECM protein and cytokine involved in various physiological and pathological processes, particularly bone remodeling. As a member of the small integrin-binding ligand N-linked glycoprotein (SIBLING) family, OPN plays a crucial role in regulating cellular interactions in bone tissue and influences the differentiation of bone marrow cells into osteoblasts and osteoclasts, essential for bone formation and resorption [70]. Additionally, its role is especially important in response to unloading–induced changes, where it helps regulate the balance between osteoblast and osteoclast activity [71]. Our study observed that protein production peaked on the 5th day of osteodifferentiation and subsequently declined during prolonged cultivation, lasting up to 20 days. These findings align with the results reported by Juhásová et al., who examined OPN expression in MSCs derived from miniature pigs [72]. Their research demonstrated an increase in OPN expression on the 7th day of differentiation, followed by a gradual decline with extended cultivation up to 21 days. Similarly, there is substantial evidence to support the notion that OPN protein levels or its gene expression are elevated during the early stages of osteogenic differentiation in MSCs or MSC-like cells. Other studies corroborate this trend [73,74]. However, a decline in OPN expression with prolonged differentiation has also been documented [72,75]. They reported gene expression decrease in human MSCs after one week or four weeks, respectively, of differentiation using RT-PCR analysis. The results revealed that OPN could play an important role in the early stages of osteogenic differentiation, with its expression decreasing as the differentiation process progresses.

Actin, one of the main cytoskeletal components in eukaryotic cells, exists as globular actin (G-actin) and filamentous actin (F-actin). F-actin filaments contain G-actin subunits and undergo constant assembly and disassembly. Actin cytoskeleton remodeling plays a crucial role in various cellular processes, including deformation, proliferation, migration, signaling, and apoptosis [76,77]. Changes in the actin network generate mechanical signals that cells respond to, transforming mechanical forces into biochemical signals. The contractility of actin filaments can stretch associated proteins, exposing new binding sites and activating signaling proteins through phosphorylation. The dynamics influence the osteogenic differentiation of BMMSCs, although the exact mechanisms remain unclear. In particular, actin reorganization is regulated by key transcriptional factors such as p53, NKX2.5, and TAZ [78,79]. We initially observed a marked increase in the intensity of β-actin fibrils on the 10th day of differentiation. However, following this peak, there was a rapid decline in intensity, which was notably lower than in the control cells. Control cells showed a much stronger intensity of β-actin fibrils, which was consistent with an increase in the number of cells. In contrast, although cell remodeling was significantly more prominent in differentiated cells, the chosen visualization technique did not adequately capture this level of structural change.

The limitation of this study is that it was conducted on samples from only two patients. Expanding the sample size and including diverse sources of dental tissue could provide a more comprehensive characterization of specific osteogenic protein expression patterns. Additionally, the heterogeneity of the differentiated cell population may influence the expression level of osteogenic proteins, as the analyzed cells comprised a mixture of stem cells, progenitors, osteoblasts, and osteocytes. In this context, single-cell analysis, along with more comprehensive approaches, would be valuable for a deeper understanding of the osteogenic differentiation of DPSCs. Elucidating the mechanisms underlying the therapeutic effects of DPSCs remains a significant challenge that must be addressed before stem cell-based therapies can be translated into clinical practice.

## 5. Conclusions

Our work provides new information on the dynamic differentiation process of DPSCs into bone cells. The results indicate that DPSC osteodifferentiation involves multiple strictly time-dependent and highly regulated pathways. Some osteogenic proteins, such as COL1A1 and OPN, are involved in the osteodifferentiation process, particularly in its early stages, while BMP9, DSPP, and MMP8 increase their expression in the later stages. APOA2 and COL1 proteins are among the main proteins forming the ECM during the osteodifferentiation of DPSC cells. We demonstrate the ability of DPSC cells to differentiate into bone cell precursors and mature bone cells, thus confirming their potential for use in regenerative medicine. Understanding the molecular mechanisms and cellular regulations of stem cell differentiation to bone cells would shed light on the osteogenesis procedure and support the use of adult DSCs in the field of regenerative medicine for bone tissue.

## Data Availability

The datasets generated during and/or analyzed during the current study are available from the corresponding author upon reasonable request.

## Supplementary Materials

The following supporting information can be downloaded at: https://www.mdpi.com/article/10.3390/biology14030257/s1, Figure S1: Alkaline phosphatase staining of control and osteodifferentiated dental pulp stem cells (DPSCs). Figure S2: Immunocytochemical staining of osteogenesis markers in control cells.

## Abbreviations

The following abbreviations are used in this manuscript:

ALP	Alkaline phosphatase
APOA2	Apolipoprotein A2
BMMSCs	Bone marrow mesenchymal stem cells
BMPs	Bone morphogenic proteins
COL1	Collagen type 1
DFPCs	Dental follicle precursor cells
DMP1	Dentin matrix protein 1
DPBS	Dulbecco’s phosphate-buffered saline
DPSCs	Dental pulp stem cells
DSCs	Dental stem cells
DSPP	Dentin sialophosphoprotein
ECM	Extracellular matrix
MEPE	Stromal extracellular phosphoglycoprotein
MMP8	Matrix metalloproteinase 8
MSCs	Mesenchymal stem cells
NCPs	Non-collagenous mineral binding proteins
OCN	Osteocalcin
OPN	Osteopontin
PDLCs	Periodontal ligament stem cells
PFA	Paraformaldehyde
PHALL	Phalloidin
RT	Room temperature
SCAP	Stem cells from the apical papilla
SHEDs	Stem cells from human exfoliated deciduous teeth
