# Supplementary material for: Temporal Profiling of Cellular and Molecular Processes in Osteodifferentiation of Dental Pulp Stem Cells
Source: Biology (Basel). 2025 Mar 4;14(3):257. doi: 10.3390/biology14030257 (PMC11939960; doi:10.3390/biology14030257)
Supplement: Supplementary file 1 [file biology-14-00257-s001.zip › biology-3498712-supplementary.pdf]

biology

MDPI

Supplementary material

Temporal Profiling of Cellular and Molecular Processes in Osteodifferentiation of Dental Pulp Stem Cells

Bibiána Baďurová [1,2], Kristina Nystøl [1,†], Terézia Okajček Michalič [1], Veronika Kucháriková [1,2], Dagmar Statelová [3], Slavomíra Nováková [1], Ján Strnádel [1], Erika Halašová [1] and Henrieta Škovierová [1,*]

1 Biomedical Centre Martin, Jessenius Faculty of Medicine in Martin, Comenius University in Bratislava (JFM CU), Malá Hora 4C, 036 01 Martin, Slovakia; badurova25@uniba.sk (B.B.); kristina.nystol@so-hf.no (K.N.); tereza.okj@gmail.com (T.O.M); kucharikova14@uniba.sk (V.K.); slavomira.novakova@uniba.sk (S.N.); jan.strnadel@uniba.sk (J.S.); erika.halasova@uniba.sk (E.H.)

2 Department of Medical Biochemistry, JFM CU, Malá Hora 4D, 036 01 Martin, Slovakia

3 Department of Stomatology and Maxillofacial Surgery, University Hospital in Martin and JFM CU, Kollárova 2, 036 01 Martin, Slovakia; dagmar.statelova@uniba.sk

* Correspondence: henrieta.skovierova@uniba.sk; Tel: +421-432-633-904

† Current address: Sykehuset Østfold Kalnes, Kalnesveien 300, 1714 Grålum, Norway

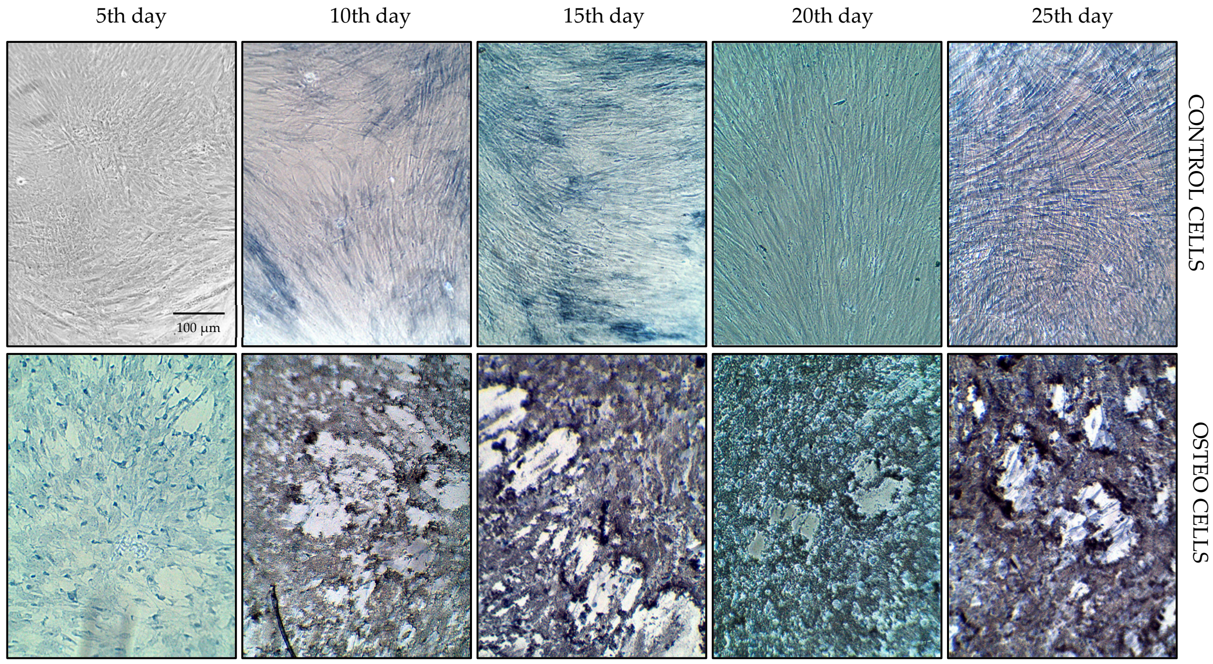

Figure S1. Alkaline phosphatase staining of control and osteodifferentiated dental pulp stem cells (DPSCs) in a time dependent manner in 25 days since the beginning of osteodifferentiation. Changes in expression level of alkaline phosphatase (ALP) was monitored by light microscopy.

 https://doi.org/10.3390/biology14030257

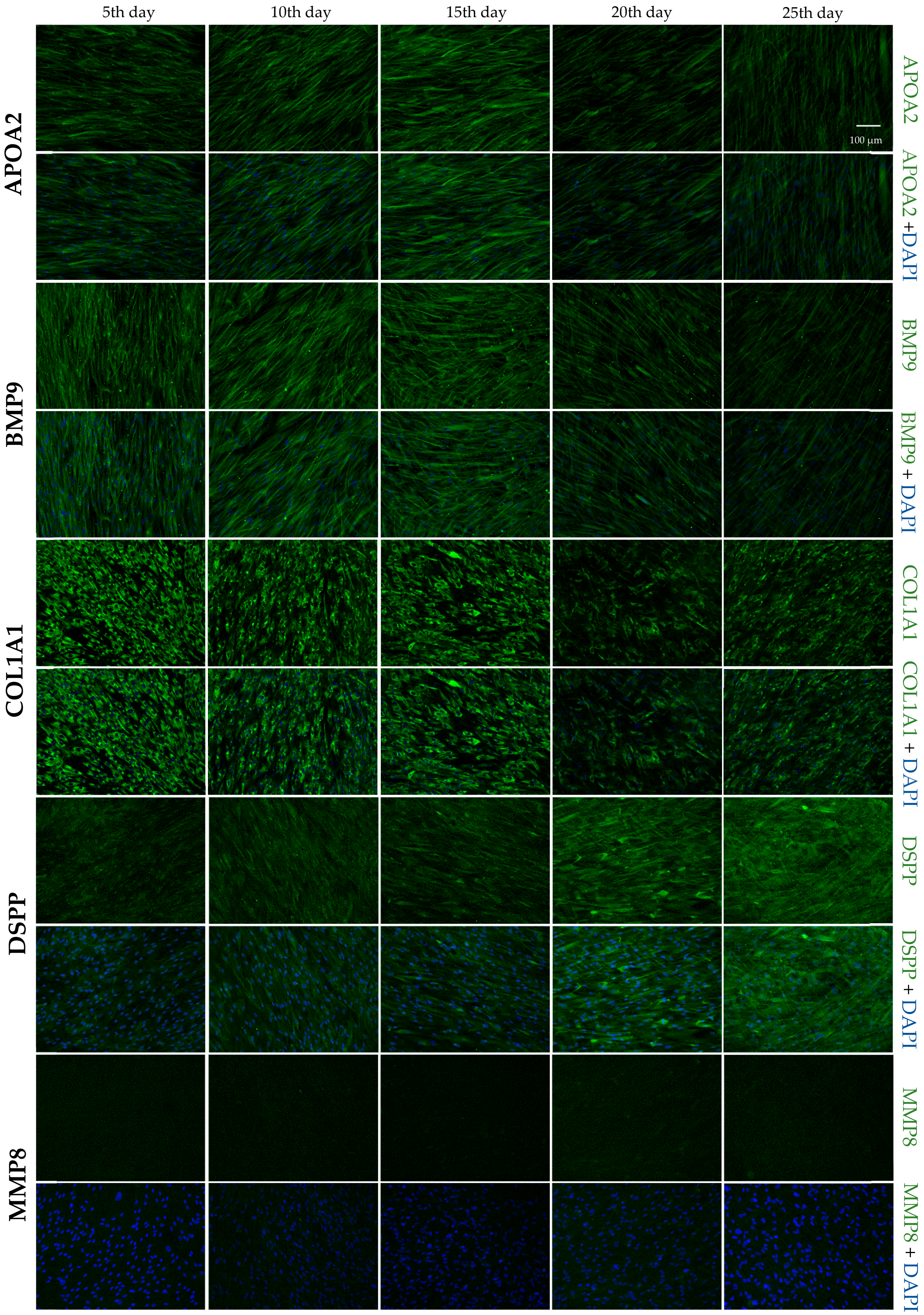
5th day
10th day
15th day
20th day
25th day
APOA2
BMP9
COL1A1
DSPP
MMP8
APOA2
APOA2+DAPI
BMP9
BMP9 + DAPI
COL1A1
COL1A1 + DAPI
DSPP
DSPP + DAPI
MMP8
MMP8 + DAPI
100 μm

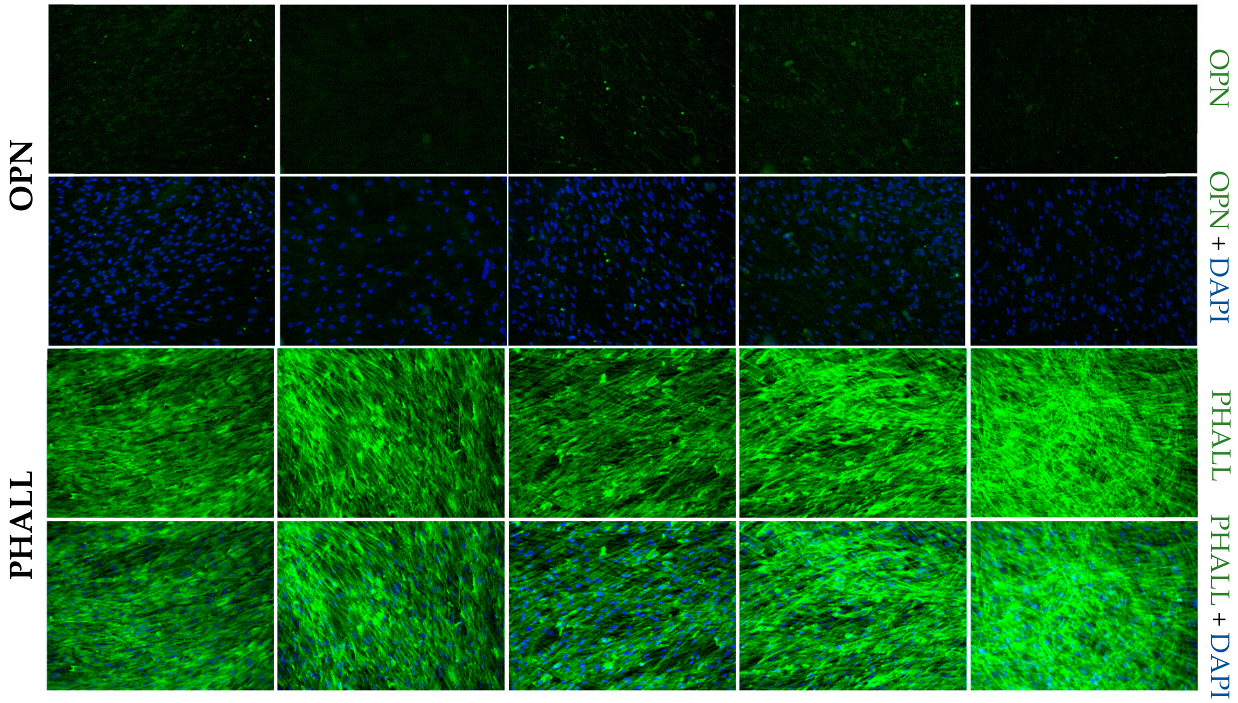

Figure S2. Immunocytochemical staining of osteogenesis markers was performed using fluorescent antibodies (green) at 25 days following the initiation of osteodifferentiation in dental pulp stem cells (DPSCs). This figure displays control cells stained on the 5th, 10th, 15th, 20th, and 25th day with specific fluorescent antibodies targeting apolipoprotein A2 (APOA2), bone morphogenetic protein 9 (BMP9), collagen1 A1 (COL1A1), dentin sialophosphoprotein (DSPP), matrix metalloproteinase 8 (MMP8), and osteopontin (OPN). Actin filaments of the cytoskeleton were labeled with an antibody against phalloidin (PHALL), and nuclei were stained with DAPI (blue). The results of each protein staining were visualized by fluorescence microscopy.
